# Supplementary material for: Acceptance and compliance with micronutrient powder (MNP) among children aged 6–23 months in northern Nigeria
Source: PLOS Glob Public Health. 2022 Oct 17;2(10):e0000961. doi: 10.1371/journal.pgph.0000961 (PMC10022258; doi:10.1371/journal.pgph.0000961)
Supplement: S1 Dataset — (ZIP) [file pgph.0000961.s008.zip › S8_Dataset/Spot Checks.zip!/Spot Checks/05072015_A_SO_AS_2_SC.pdf]

SPOT CHECK RECORDING FORM

State: Adamawa **LGA: ____Song___ Community: _Bongo_**

Name of caregiver (female): Talatu Babagari

Name or caregiver (male): ………………………

Sex of index child: _Male___ **Age of index child (in months): _22 months_______**

Spot Check Information

1. **Date of MNP distribution to household** (circle option below based on LGA name above)**:**
   - **Kebbi:** BirninKebbi&Jega LGA: **June 16**
   - **Kebbi:** Danko-wasagu: **June 17**
   - **Adamawa:**Song, Ganye: **(June 18)**
   - **Adamawa:**Guyuk **June 19**
2. **Date of Spot Check Observation: ____5/7/2015_____**
3. **Number of sachets remaining**(Ask the caregiver to show you and count the specific number of sachets remaining)**:**

   42

4. **Number of sachets that should remainusing one MNP sachet per day** (Analyst only)**:**

   44

5. **Number of sachets remaining / number of sachets should be remaining**(Analyst only)**:**

   _____42____ / ___44______ = _95.5___ %

Probes

Question 1. You have **XX** MNP sachets remaining. Explain how easy or difficult it was to use the MNP product on a daily basis for your child 6 – 23 months? (Circle one answer)

Very Difficult **Difficult** **(Easy)** **Very Easy**

Question 2. Describe any specific challenges that you faced that made the MNP difficult to use on a daily basis?
I wouldn't say it is a challenge but unsealing the sachet is somehow difficult for me. Some days I use my teeth and the powder may pour on the ground.

Question 3. Describe specific aspects of the MNP product that you or your child enjoyed that made it easier to use on a daily basis?

The fact that the product does not taste bitter is very satisfactory to me because our children usually reject anything that is bitter.

Question 4. What questions do you have about the MNP based on your experience using it?

- Should I or can I use the MNP and still give my child other syrup like paracetamol or cough syrup. I wouldn't want other syrup to reduce the efficacy of the MNP. I want to be sure

Question 5. What specific recommendations do you have that we can do as a program to make the MNP easier to use on a daily basis?

Yes the sachet should be together to unseal, and also the size should be increase a little bit. Aside from these two comments I have no other concern
